# Genetic heterogeneity of chicken anemia virus isolated in selected Egyptian provinces as a preliminary investigation

**DOI:** 10.3389/fvets.2024.1362219

**Published:** 2024-05-22

**Authors:** Sara Abdel-Mawgod, Ali Zanaty, Mohamed Elhusseiny, Dalia Said, Abdelhafez Samir, Moataz M. Elsayed, Osama Mahana, Mahmoud Said, Ahmed M. Hussein, Heba M. Hassan, Abdullah Selim, Momtaz A. Shahien, Karim Selim

**Affiliations:** ^1^Reference Laboratory for Veterinary Quality Control on Poultry Production, Animal Health Research Institute, Agriculture Research Center, Giza, Egypt; ^2^Division of Pharmacology and Toxicology, Department of Pharmaceutical Sciences, University of Vienna, Vienna, Austria

**Keywords:** CAV, full genome sequence, pathogenicity, Egypt, PCR

## Abstract

Chicken anemia virus (CAV) is a widespread and economically significant pathogen in the poultry industry. In this study 110 samples were collected from various poultry farms in selected Egyptian provinces during 2021–2022 and were tested against CAV by Polymerase Chain Reaction (PCR), revealing 22 positive samples with 20% incidence rate. Full sequence analysis of five selected CAV strains revealed genetic variations in VP1, VP2, and VP3 genes. Phylogenetic analysis grouped the Egyptian strains with reference viruses, mainly in group II, while vaccines like Del-Rose were categorized in group III. Recombination events were detected between an Egyptian strain (genotype II) and the Del-Rose vaccine strain (genotype III), indicating potential recombination between live vaccine strains and field isolates. To evaluate pathogenicity, one Egyptian isolate (F883-2022 CAV) and Del-Rose vaccine were tested in Specific Pathogen Free (SPF) chicks. Chicks in the positive group displayed clinical symptoms, including weakness and stunted growth, with postmortem findings consistent with CAV infection. The vaccine group showed milder symptoms and less severe postmortem changes. This study provides important insights into the genetic diversity of CAV in selected Egyptian poultry farms showing recombination event between field strain and vaccine strains, highlighting the need for advanced vaccination programs, especially for broilers.

## GRAPHICAL ABSTRACT

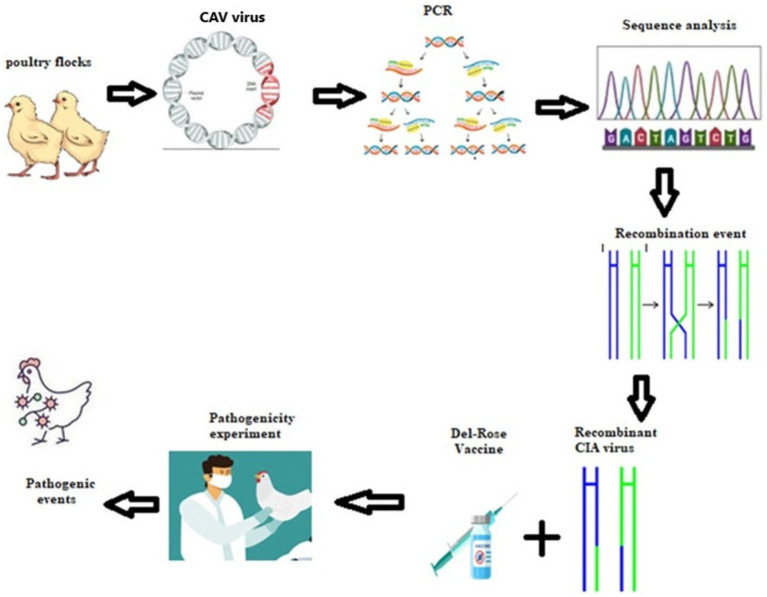

## 1 Introduction

Since its initial detection in Japan in 1979, CAV is known to be prevalent in countries with significant chicken production. This viral infection, commonly referred to as CAV, has substantial economic implications for the poultry industry, causing immunosuppression and resulting in substantial financial losses (1). In Egypt, the first CAV case was detected by El-Lethi (2) in a commercial chicken farm in 1990. Consequently, numerous studies have reported the circulation of CAV in chicken farms among Egypt. Therefore many studies was done to molecular detect and characterize of CAV as a trail to control of CAV (3).

CAV can be transmitted both vertically and horizontally, and the infection can manifest as either clinical or subclinical in affected birds. Typically, young birds less than 2–3 weeks old may exhibit clinical symptoms such as anemia, stunted growth, and increased mortality, as well as post-mortem findings including thymus and bone marrow atrophy and subcutaneous hemorrhages (4). Proteolytic enzymes are classified according to their catalytic domains into four main groups which are cysteine, serine, aspartic and metalloproteinases (5, 6). Proteases enzymes play a pivotal role in both the infection and potential treatment of CAV. During CAV infection, protease enzymes are crucial for the processing of viral polyproteins, as observed in other similar viruses (7). Upon entering the host cell, the virus introduces its genetic material, which includes a polyprotein that requires division into functional viral proteins. This crucial cleavage process is carried out by protease enzymes, and it plays a pivotal role in the replication of the virus and the formation of new viral particles (8). Researchers are exploring the development of antiviral drugs targeting these protease enzymes, effectively inhibiting the cleavage process and preventing the formation of mature, infectious CAV particles (9).

CAV belongs to the Gyrovirus genus within the Circoviridae family and stands as its sole member. It possesses a non-enveloped structure and features a negative-sense, single-stranded, circular DNA genome with a length ranging from approximately 2,298 to 2,319 nucleotides (10). Within its genome, three open reading frames partially overlap with each other, responsible for encoding the viral proteins VP1, VP2, and VP3, as described by (11). VP1 and VP2 are key components of protective antigen proteins that interact with neutralizing antibodies. Additionally, the VP1 gene plays a role in viral replication and contributes to the pathogenicity of the virus (12). CAV’s amino acid composition is remarkably consistent, except for VP1, which exhibits variability in specific regions, notably within amino acid positions 139 through 151. As a result, the VP1 gene is the primary target for the genetic characterization of CAV strains (13). Vp3 is identified as a virulence factor that triggers apoptosis in susceptible chicken lymphoblastoid T and myeloid cells (14). Notably, apoptin has been found to induce apoptosis in various human cancer cell lines independently of the p53 protein (15). Moreover, researchers have explored apoptin’s potential in gene therapy using an adenoviral vector, and it has shown promise by reducing tumors in mice without significant side effects (1). The ongoing research and reviews on apoptin’s potential as a cancer therapeutic continue (16).

In the context of viral proteases, their ability to modulate their proteolytic activity can have both detrimental and beneficial effects on the virus. When the virus enters a host cell, it releases its genetic material, including a polyprotein that must undergo cleavage to form functional viral proteins. This cleavage process is orchestrated by protease enzymes and is indispensable for viral replication and the assembly of new viral particles (17).

Scientists are actively exploring the development of antiviral drugs that target these protease enzymes, effectively inhibiting the cleavage process and preventing the maturation of infectious CAV particles (9).

Globally, the analysis of the VP1 gene’s nucleotide sequence has led to the recognition and reporting of four distinct genogroups/genotypes, denoted as I, II, III, and IV (18). Furthermore, within the VP1 protein, specific genetic markers exist that facilitate the differentiation between vaccine-derived and field strains (19). The CAV genome has been documented to undergo recombination events, potentially giving rise to the emergence of novel genotypes (20). For the fact of good monitoring is the magic key to perfect controlling.

### 1.1 Aim of study

The study aimed to conduct molecular identification and characterization of circulating CAV strains in selected Egyptian farms, along with a comprehensive analysis of their genetic diversity, recombination events, and pathogenicity, providing insights into the coexistence of field and vaccine-derived strains and their implications for poultry health management.

## 2 Materials and methods

### 2.1 Sampling

Between 2021 and 2022, a total of 110 poultry farms, encompassing broilers, breeders, and layers, distributed across 12 different governorates, were the source of sampled materials. Tissue samples from 5 to 10 birds were collected from apparent health farm, while the infected or suspected cases were collected from infected farms.

These samples were derived from various tissues, including the liver, spleen, thymus, and bone marrow. To prepare the tissue samples, they were meticulously ground using a mortar and pestle in a solution of PBS, which included an antibiotic mixture (comprising 1000 I.U. penicillin per milliliter and 1 mg of streptomycin sulfate per milliliter), resulting in a 20% tissue homogenate. This homogenate underwent a cycle of freezing and thawing three times and was subsequently centrifuged at 3000 revolutions per minute for 20 min. The resulting supernatant was carefully transferred into new tubes for use in PCR and genetic characterization.

### 2.2 PCR detection of CAV

DNA extraction from the examined tissue homogenates was performed utilizing the QIAamp DNA extraction kit (Qiagen, Germany, Cat. No 51304), Quality of extracted DNA was measured by Nabi Nanodrop spectrophotometer |(MicroDigital Co., Ltd., Korea) and DNA concentrations of positive samples were listed in (Supplementary Table 1) to amplify a 675 bp DNA fragment of the Vp1 gene (21), the oligonucleotide primers 5’-GAC TGT AAG ATG GCA AGA CGA GCT C-3′ and 5’-GGC TGA AGG ATC CCT CAT TC-3′ were employed, spanning nucleotides from 823 to 1498, numbering is corresponding to the Del-Rose strain (GenBank AF313470) (3). The PCR assay was conducted using the Emerald Amp®GT PCR master mix (Cat No. RR310A) in a final volume of 50 μL, with the following components: 25 μL of master mix, 15 μL of PCR grade water, 2 μL for each primer, and 5 μL of templates. The amplification process followed these conditions: one initial cycle with a denaturation step at 95°C for 15 min, followed by 40 cycles at 95°C for 45 s, 56°C for 45 s, and 72°C for 1 min, with a final extension cycle at 72°C for 10 min. The resulting amplification products were assessed through electrophoresis on a 1.5% agarose gel, stained with ethidium bromide, and visualized under a transilluminator. The size of the PCR products was verified by comparing them against a DNA ladder (GelPilot® 1 kb Ladder, Qiagen, Germany). Positive samples were subjected to full genome sequence amplification through PCR, using the designed primers in Table 1, following the same conditions described above.

**Table 1:** Designed primers used for full genome sequence amplification through PCR.

	Primers sequences	Product size	References
F1-1	5’ CCGAGTGGTTACTATTCCATCACC 3’	668 bp	(22)
R1-668	5’ CAGCGATAGAGTGATTGTAATTCC 3’		
F2-502	5’ TTCAGGCCACCAACAAGTTCACGG 3’	698 bp	
R2-1200	5’ CCGCAATCAACTCACCGGCGATGG 3’		
F3-1350	5’ ATGCAGCCCACGGACTCTTGCCGG 3’	900 bp	
R3-2250	5’ CTGGGGGGGAATCCCCCCCAGGGG 3’		

### 2.3 Full genome nucleotide sequence and phylogenetic analysis

From the pool of positive CAV samples, we handpicked five isolates for complete genome sequencing. To prepare for sequencing, the PCR fragments that cover the full genomes were purified using the QIAquick® Gel Extraction Kit Cat No. 28704 (Qiagen, Germany) in adherence to the manufacturer’s guidelines. The sequencing was performed directly, employing the ABI PRISM® BigDye® Terminators v3.1 Cycle Sequencing Kit Cat No. 4336917 (Applied Biosystems, Foster City, CA, USA), and the data were generated using the ABI PRISM® 3,130 genetic analyzer (Applied Biosystems) with 80 cm capillaries.

To process and analyze the sequences, we used SeqScape® Software Version 2.5 (Applied Biosystems) for editing. Creating consensus sequences and trimming alignment were carried out using Bioedit software (version 7) (23) with the Clustal W method (24). We constructed a phylogenetic tree with MEGA7 software (25) making comparisons with reference viruses from GenBank and other Egyptian strains. Lastly, we determined the identity of all the viruses using Bioedit software.

### 2.4 Recombination analysis using RDP

To identify recombination events, we utilized RDP v4.5 software and assessed the presence of such events using various analytical tools, including RDP, GENECONV, BootScan, MaxChi, Chimaera, SiScan, and 3Seq. A *p*-value of less than or equal to 0.05 was employed to validate the detection of recombination areas. For each recombination event, we precisely determined the starting and ending breakpoints. Furthermore, when identifying recombination areas, we also investigated the relationships between the identified parent sequences involved in each recombination event (26).

### 2.5 Isolation and pathogenicity evaluation of CAV isolate

For isolation, 1-day-old SPF chicks (10 chicks/ group) were intramuscularly inoculated by 0.1 mL of (F883-2022 CAV) positive samples (27). To assess and compare the pathogenicity of the field strain (F883-2022 CAV) and the CAV vaccine (Del-Rose vaccine), we conducted an experiment using forty-five one-day-old SSPF chicks. These chicks were divided into three groups, each consisting of 15 chicks. In the first group, labeled Group 1, the chicks were intramuscularly inoculated with 0.1 mL of the selected field strain, F883-2022 CAV. The second group, referred to as Group 2, received an intramuscular inoculation of the Del-Rose vaccine (Cevac® Circomune L). The third group served as the negative control, and these chicks were not subjected to any inoculation. The three groups were kept in chicken isolators in experimental research center BSL3-Animal Health Research Institute.

After a designated period, specifically 21 days, a subset of the birds was humanely euthanized. Subsequently, postmortem examinations were conducted, and PCR tests were performed to evaluate the effects and outcomes of the inoculations. This analysis aimed to provide a clear and detailed understanding of the pathogenicity and responses of the chicks to the field strain and the CAV vaccine.

## 3 Results

### 3.1 Clinical signs and post-mortem findings

One hundred and ten (110) tissue homogenates (liver, thymus and spleen) were collected from broilers, layers and breeder poultry farms located in selected Egyptian provinces during (2021 and 2022). They showed generalized weakness, depression and marked stunted growth with a moderate mortality rate. The necropsy findings were congested and fragile bone marrow, markedly atrophied thymus glands, atrophied bursa of fabricius and enlarged liver and spleen.

### 3.2 Results of PCR detection of CAV

Amplifying the DNA extracted from 110 tissue samples resulted in the identification of 22 positive DNA bands of the correct size, measuring 675 bp, through conventional PCR. This yielded an overall infection rate of 20%. Notably, the highest incidence of CAV was observed in the governorates of Beniseuif, Monifia, and Sharkia, with positivity rates of 50, 50, and 32%, respectively. On the other hand, Cairo and Matrouh showed no detection of CAV. Among the 22 positive CAV samples, the distribution by poultry type was as follows: 14 samples from broilers, 4 samples from layers, and 4 samples from breeders. The incidence rates for these groups were 64, 18, and 18%, respectively (Table 2).

**Table 2:** Incidence of CAV infection among poultry farms in12 different Egyptian Provinces.

Provinces	No. of farms	No. of positives farms	Percentage of positivity	Flocks breeds		
				Layers	Broiler	Breeders
Dakahlia	11	1	9%	–	–	1
Ismalia	11	2	18%	1	1	–
Sharkia	28	9	32%	2	7	–
Kafr El sheikh	3	–	–	–	–	–
Giza	8	1	12.5%	–	1	–
Alex	14	4	28.6%	1	3	–
Cairo	2	–	–	–	–	–
Mars matroh	4	–	–	–	–	–
Mienya	5	–	–	–	–	–
Beniseuif	2	1	50%	–	1	–
Monifia	4	2	50%	–	–	2
Behera	18	2	11%	–	1	1
Total No	110	22	20%	4 (18%)	14 (63.6%)	4 (18%)

### 3.3 Nucleotide and amino acid sequence analysis

The full genome sequences of five selected CAV strains in this study were compared with other CAV reference strains in GenBank by multiple alignments with the ClustalW included in Bioeditsoftware. The nucleotide sequence analysis showed that five (F883/2022, F894/2022, F899/2022, F233/2021, 2/2021) viruses were closely related to each other with identity 98–100%. The amino acid sequence of the five CAV strains showed motif as T89, L125, Q141, and E144 strains While f233 showed substitution I75 like other reference strains as BD-3 TR20 and LF4and other Egyptian strains which belong to group II Egypt/Cai1/2015 and 2/2021 strain shows different substitution as L75. All strains also showed substitution Q22, T89 and V157 (Table 3). VP2 amino acid sequence also showed substitution at residues V153 and E 175 for the three CAV strains, in addition to T 180 S in all isolates like other Egyptian strains (CAV/CA1 and CAV/GZ2) and reference 98D06073-USA while VP3 showed only one substitution C118 in all of them.

**Table 3:** Amino acid substitutions comparison between five Egyptian CAVs and other reference CAV.

Virus name	Accession number	Country	22	75	89	97	125	139	141	144	157
GD-1-12-China	JX260426	China	H	V	T	M	L	K	Q	E	M
TJBD40-China	AY846844	China	–	–	–	–	–	–	–	–	V
98D02152-USA	AF311892	USA	–	–	–	–	I	–	–	–	V
3-1P60-Malaysia	AY040632	Malaysia	–	–	–	–	I	–	–	–	–
A2-Japan	AB031296	Japan	–	–	–	–	I	–	–	–	–
LF4-China	AY839944	China	–	I	–	–	–	Q	–	Q	V
BD-3-Bangaldesh	AF395114	Bangaldesh	–		–	L	I	Q	–	Q	V
98D06073-USA	AF311900	USA	–	I	–	L	I	Q	–	Q	V
704-Australia	U65414	Australia	–	I	–	L	I	Q	–	Q	V
TR20-Japan	AB027470	Japan	–	I	–	L	I	Q	–	Q	V
CAV/Egypt/Cai1/2015	MG827098	Egypt	Q	I	–	L	I	Q	–	Q	V
CAV/Egypt/Giz2/2016	MG827099	Egypt	Q	–	–	L	I	Q	–	Q	V
CAV/Egypt/Sha4/2017	MG827100	Egypt	–	–	–	–	I	–	–	–	V
Del-Ros/USA	AF313470	USA	H	V	T	M	I	K	Q	E	V
Cux-1-USA	NC001427	USA	H	V	T	M	I	K	Q	D	V
26P4 CAV	D10068	Netherland	H	V	T	M	I	K	Q	E	M
CAV/CH/EGYPT-2/2021	OQ376290	Egypt	Q	L	T	M	I	K	Q	E	V
CAV/CH/EGYPT-F233/2021	OQ376291	Egypt	Q	I	T	M	I	K	Q	E	V
CAV/CH/EGYPT-F883/2022	OQ376292	Egypt	Q	V	T	M	I	K	Q	E	V
CAV/CH/EGYPT-F894/2022	OQ376293	EGYPT	Q	V	T	M	I	K	Q	E	V
CAV/CH/EGYPT-F899/2022	OQ376294	EGYPT	Q	V	T	M	L	K	Q	E	V

### 3.4 Phylogenetic analysis of CAV genome

**Figure 1:**
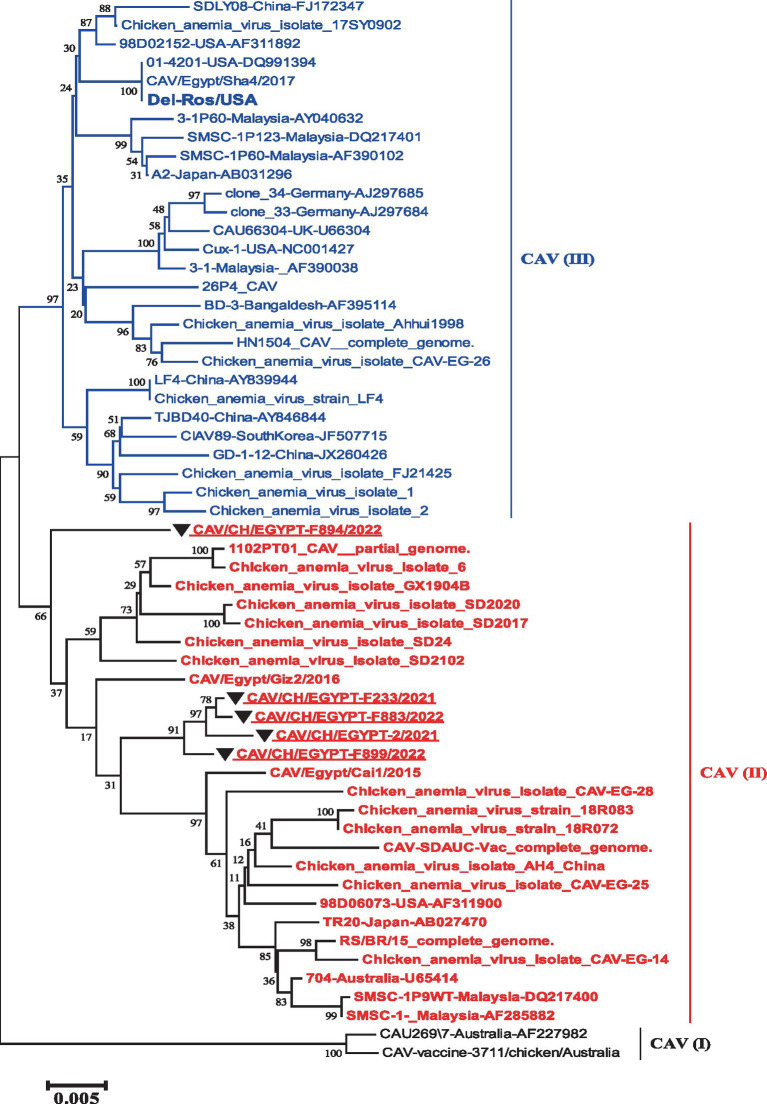
Nucleotide based phylogenetic tree of the complete genome of Egyptian CAV, where the five strains involved in this study were grouped into one group II with reference strain. The phylogenetic tree was computed in the MEGA 7 software using neighbor-joining method with 1000 bootstrap replicates using Kimura-2 parameter and nucleotide substitution model.

The phylogenetic tree of the complete genome revealed the presence of three groups that were as I, II and III. The five strains involved in this study were grouped into group II with reference strains (TR20 and Australia 704). While the vaccines like (Del-Rose) were grouped into group III (Figure 1).

### 3.5 Recombination analysis using RDP

**Figure 2:**
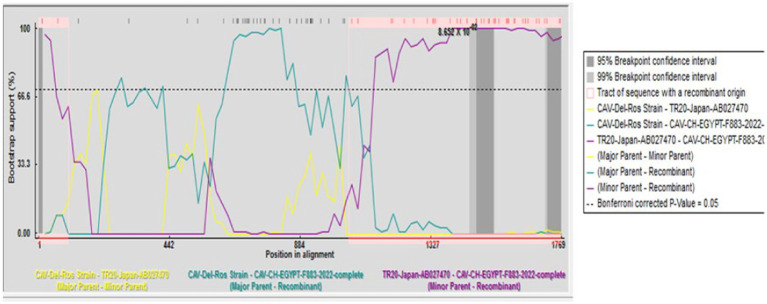
Recombination analysis displaying possible recombination events predicted to have occurred in the CAV genome segment of “CAV-CH-EGYPT-F883-2022” with TR20-Japan-AB027470 as minor parent recombinant and CAV-Del-Rose strain as major parent recombinant.

A recombination event was observed between the strains under study CAV-CH-EGYPT-F883-2022 (genotypeII), and CAV-Del-Rose vaccine strain (genotype III), while no recombination event was detected in the other strains (Figure 2).

### 3.6 Pathogenicity evaluation of (F883-2022 CAV strain) and (Del-Rose vaccine)

**Figure 3:**
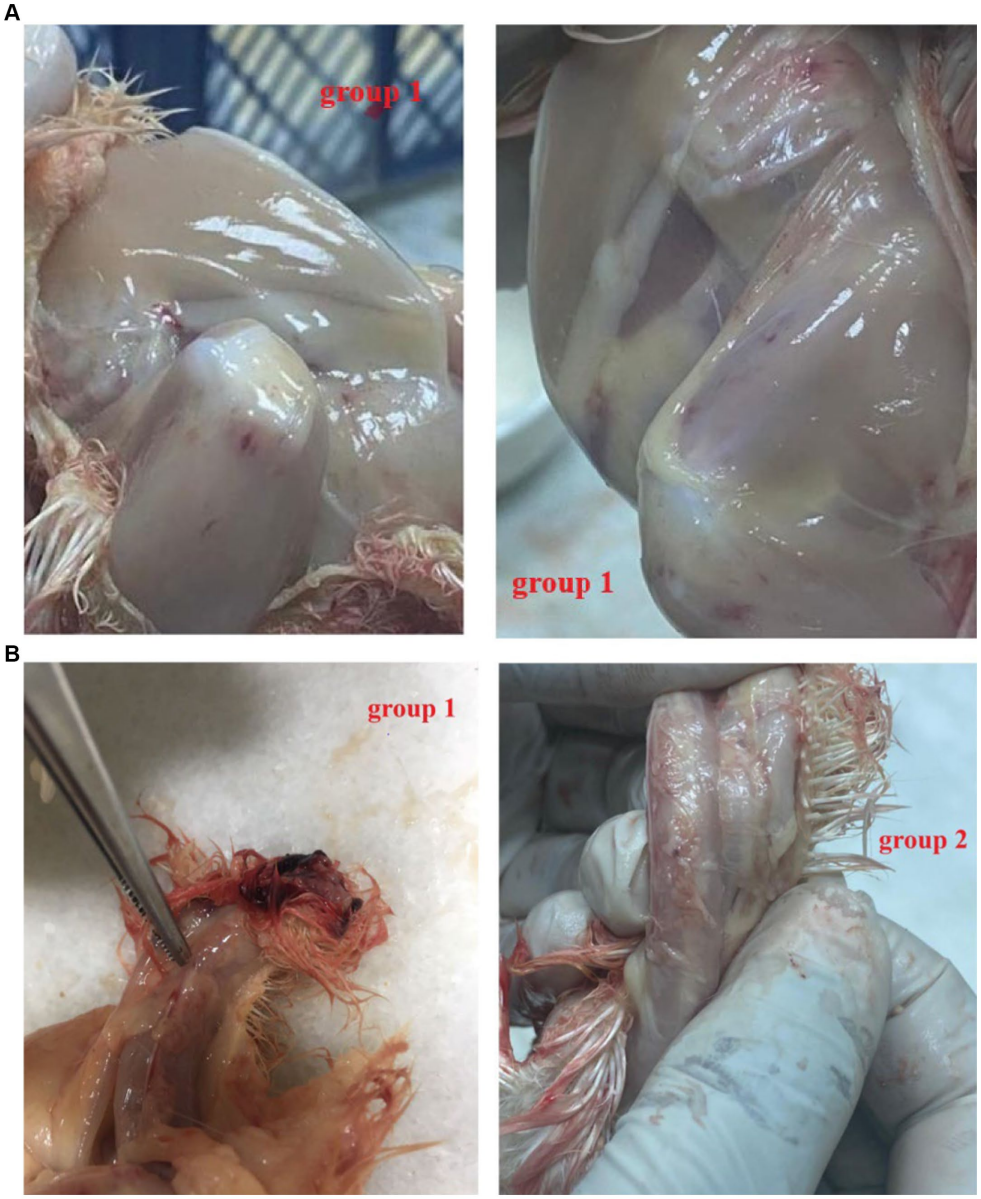
**(A)** *Post mortem* lesion after 21 days post-infection. Note that Group 1 (F883-2022 strain): hemorrhagic thigh muscle (++). **(B)** *Post mortem* after 21 days post-infection. Group 1 (F883-2022 strain): Congested thymic lobules (++), Group 2 Del-Rose CAV vaccine: Congested thymic lobules (+).

All the chicks in the positive group 1 developed the expected clinical symptoms including weakness, ruffled feathers and stunted growth after 7–10 days, while the chicks in group 2 and the negative group did not develop any clinical symptoms of CAV. In postmortem examination, both groups (1 and 2) generally showed congested and fragile bone marrow, thymus gland atrophy and enlarged liver and spleen. While group 1 showed post-mortem more severe than group 2 (Figures 3A,B). The CAV was detected in Group 1 and Group 2 with positive percent of 80 and 95% by PCR (Table 4).

**Table 4:** *Post mortem* finding of F883-2022 CAV strain and Del-Rose CAV vaccine virus (+): mild, (++): moderate and (+++): sever.

Day	PM	Group 1 F883-2022 strain	Group 2 Del-Rose CAV vaccine	Group 3 Negative group
21\PI	Pale carcass	(++)	(+)	(−)
	Congested thymic lobules	(++)	(+)	(−)
	Pale liver	(+++)	(−)	(−)
	Enlarged liver	(++)	(+)	(−)
	Fragile BM	(++)	(−)	(−)
	Hemorrhagic thigh muscle	(++)	(−)	(−)

## 4 Discussion

CAV, a pathogen that adversely affects commercial poultry, is known to induce severe immunosuppression in chickens. The first documented occurrence of CAV in Egypt dates back to 1990 (2). The virus infection is primarily characterized by lymphoid atrophy and aplastic anemia, particularly among young chicks. Throughout the surveillance period from 2021 to 2022, a systematic collection of samples from poultry farms in various governorates across Egypt was conducted to evaluate the prevalence of CAV infection. A total of 110 samples, representing broilers, layers, and breeders’ farms from 12 different governorates, PCR tested for CAV infection. Twenty-two samples were found to be positive, indicating an overall infection rate of 20%.

Notably, the regions with the highest incidence of CAV were Beniseuif, Monifia, and Sharkia, with positive rates of 50, 50, and 32%, respectively. Conversely, governorates like Kafr El Sheikh, Cairo, Mars Matroh, and Menya exhibited no evidence of CAV presence. These results align with a previous study by Abdel-Mawgod et al. (22), which suggested a widespread distribution of CAV among chicken flocks in Sharkia governorate, along with recent occurrences in Beniseuif and Monifia governorates. Further analysis of the incidence of CAV according to flock breed revealed that out of the 20 positive samples, four were from layer chickens (16.7%), four were from breeders (18%), and 14 were from broilers (64%). This data suggests that broiler flocks are particularly susceptible to CAV infection, emphasizing the significance of continued monitoring and potential vaccination programs, especially in regions with a high incidence of infection.

A hypervariable region in the VP1 protein of CAV was identified and proposed that specific amino acid substitutions within this region could impact the virus’s replication rate and its ability to spread within cell cultures (28). This hypervariable region, spanning amino acid positions 139 to 151 in VP1, was also documented in previous studies (1).

Previous research has categorized CAV isolates into three distinct groups (I, II, III) based on specific amino acid residues at positions 75, 97, 139, and 144 within the VP1 protein sequence. In the current study, it was observed that three strains (F883/2022, F894/2022, F899/2022) exhibited a profile with amino acids 75 V, 97 M, 139 K, and 144E. In contrast, strain F233/2021 displayed a substitution of I75, consistent with reference strains such as BD-3, TR20, LF4, and other Egyptian strains like EGY1 and /Egypt/Cai1/2015. These findings suggest the coexistence of genotypes II and III in Egypt, possibly influenced by the misuse of live CAV vaccines (1). Additionally, the presence of a 75 L substitution in the hypervariable region of the 2/2021 strain may indicate a recombination event (29). Interestingly, the five CAVs strains have Q22 instead of H22 like other Egyptian reference strains Egypt/Cai1/2015. It was observed the presence of Q or N instead of H at position 22 of VP1 and considered this residue to be important for distinguishing CAV strains (30).

The presence of amino acids 139Q and/or 144Q in the VP1 protein significantly affected the rate of viral replication and the spread of infection in cultured cells (28). Their research demonstrated that having these two specific amino acids was linked to a reduced rate of viral spread in cell culture. However, the strains examined in this study featured amino acids 139 K and 144E in place of 139Q and 144Q. This suggests that the Egyptian CAV strains identified might possess a heightened ability to spread in cell culture. Additionally, other studies have indicated that substitutions from glutamines to lysine and glutamic acid at these positions were associated with a loss of virulence (31). VP3 is an apoptosis-inducing protein (14). On the molecular basis, our alignment revealed that R at position 118 of the VP3 is present in all Egyptian isolates. This amino acid substitution could influence the nuclear localization of the protein and the development of distinct apoptotic bodies in cell culture (29).

It was suggested that an amino acid substitution T89A in the VP1, acquired through 310 serial passages in cell culture, could be associated with the non-reactivity to a monoclonal antibody (2A9) as well as attenuation of some molecularly cloned strains (29, 32). All detected Egyptian CAVs had threonine in position 89 instead of alanine. It was suggested that this substitution is associated with attenuation (10). The previous mutation should be combined with 75I, 125 L, 141 L, and 144E to produce attenuation. One Egyptian CAV sequence (F233) displayed this combination. This pattern is closely related to vaccine strains (del-rose) (33). Vaccine persistence and reversion to virulence have also been frequently reported among other attenuated avian vaccines developed to control immunosuppressive or respiratory diseases (34, 35). Vaccine behavior could subsequently be a hazard for young chicks since it has been demonstrated that attenuated CAV strains have the potential to revert to virulent phenotypes after chicken-to-chicken transmission in the field (36, 37). This was supposed in the Egyptian CAV strain (f233) as L75I where I is related to strains among genotype II.

On the other hand, commercially live CAV vaccines are derived from field strains after serial passages in cell cultures or chicken embryos for attenuation (38). However, the level of attenuation cannot be controlled and this attenuation does not prevent vertical or horizontal transmission of the vaccines to\or between offspring (39). Next-generation vaccines, with improved stability and safety, are currently under study and development for most avian pathogens and, in the future, they will likely replace traditional vaccines (40, 41).

Recombination occurs when at least two viral genomes co-infect the same host cell and exchange genetic segments. Based on the crossing site’s structure, many viral recombination processes may be identified (42, 43). A recombination event was observed between the CAV-CH-EGYPT-F883-2022 strain (genotype II), and the CAV-Del-Rose vaccine strain (genotype I). This may indicate the presence of CAV infection while vaccinated or the continuous circulation of both live CAV vaccine and the field isolates and the recurrent recombination between them resulting in new strains carrying both genetic characteristics. These findings agree with what was reported in Egyptian broilers by (44), this information allows us to propose the possibility of a novel combination occurring between vaccine strains and field strains, giving rise to a strain derived from the vaccine or a field strain with a sequence closely resembling that of vaccines. These strains seem to be circulating in poultry farms in Egypt.

Furthermore, it has been suggested that the genetic classification of CAV strains may be associated with distinct biological characteristics of these strains. To conclusively establish the biological attributes of the genetic profiles and recombination observed in this study, additional *in vivo* pathogenicity studies should be conducted on susceptible birds within controlled and isolated conditions. This research will help further elucidate the impact and implications of these genetic variations on the pathogenicity and behavior of CAV strains in poultry populations. For further pathological evaluation, one Egyptian isolate (group 1) and del-rose vaccine (group 2) were inoculated in SPF one-day-old chicks. All the chicks in group 1 developed the expected clinical symptoms including weakness, ruffled feathers and stunted growth after 7–10 days, while the chicks in group 2 and the negative group did not develop any clinical symptoms CAV. In postmortem examination, both CAV-positive and vaccine groups generally showed identical distribution patterns in organs of tropism (thymus, spleen, liver, and bone marrow), with differences in their severity Group 1 exhibited more severe pathological changes, including congested thymic lobules and an enlarged liver when compared to Group 2, providing insights into the differing pathogenicity between the field strain and the vaccine. This agrees with the previous observation that the lesions depend on the degree of severity (13, 45).

## 5 Conclusion

According to this study, CAV is still present in Egyptian chicken farms. These findings also showed that local CAV isolates have genetic heterogeneity owing to frequent recombination between field strains and live vaccines, generating new strains with both genetic traits. Further studies are required for investigation of the pathogenicity difference among CAV strains and live vaccines strains as well as to get the relation between using live vaccines may lead to recombination events in the field CAV strains.

## Data availability statement

Information for existing publicly accessible datasets can be found in online repositories: GenBank, accession numbers: (VP1: OQ376290 to OQ376294, VP2: OQ333065 to OQ333069 and VP3: OQ333070 to OQ333074).

## Ethics statement

The animal study was approved by the Institutional Animal Care and Use Committee of the Reference Laboratory for Veterinary Quality Control on Poultry Production, Animal Health Research Institute (Code: ARCAHRI6020). The study was conducted in accordance with the local legislation and institutional requirements.

## Author contributions

SA-M: Data curation, Formal analysis, Investigation, Methodology, Software, Validation, Writing – original draft, Writing – review & editing. AZ: Data curation, Investigation, Methodology, Software, Writing – original draft, Writing – review & editing. ME: Data curation, Investigation, Methodology, Software, Writing – original draft, Writing – review & editing. DS: Data curation, Investigation, Methodology, Software, Writing – original draft, Writing – review & editing. ASa: Data curation, Investigation, Methodology, Software, Writing – original draft, Writing – review & editing. MME: Data curation, Investigation, Methodology, Software, Writing – original draft, Writing – review & editing. OM: Data curation, Investigation, Methodology, Software, Writing – original draft, Writing – review & editing. MS: Data curation, Investigation, Methodology, Software, Writing – original draft, Writing – review & editing. AH: Conceptualization, Data curation, Formal analysis, Funding acquisition, Investigation, Methodology, Software, Validation, Visualization, Writing – original draft, Writing – review & editing. HH: Data curation, Investigation, Methodology, Software, Writing – original draft, Writing – review & editing. ASe: Data curation, Investigation, Methodology, Software, Writing – original draft, Writing – review & editing. MS: Software, Writing – original draft, Writing – review & editing, Data curation, Investigation, Methodology. KS: Conceptualization, Data curation, Formal analysis, Funding acquisition, Investigation, Methodology, Project administration, Resources, Software, Supervision, Validation, Visualization, Writing – original draft, Writing – review & editing.
