# Supplementary material for: Genetic heterogeneity of chicken anemia virus isolated in selected Egyptian provinces as a preliminary investigation
Source: Front Vet Sci. 2024 May 22;11:1362219. doi: 10.3389/fvets.2024.1362219 (PMC11150715; doi:10.3389/fvets.2024.1362219)
Supplement: Supplementary file 1 [file Table_1.docx]

| Sample no. | DNA concentration (ng/µl) |
| --- | --- |
| 1 | 40 |
| 2 | 48 |
| 3 | 42 |
| 4 | 38 |
| 5 | 35 |
| 6 | 39 |
| 7 | 25 |
| 8 | 29 |
| 9 | 44 |
| 10 | 50 |
| 11 | 33 |
| 12 | 38 |
| 13 | 28 |
| 14 | 41 |
| 15 | 30 |
| 16 | 42 |
| 17 | 29 |
| 18 | 33 |
| 19 | 48 |
| 20 | 31 |
| 21 | 36 |
| 22 | 44 |

**Supplementary table 1**: DNA concentration of twenty-two CAV positive samples
